# Ferulic Acid Protects Human Lens Epithelial Cells against Ionizing Radiation-Induced Oxidative Damage by Activating Nrf2/HO-1 Signal Pathway

**DOI:** 10.1155/2022/6932188

**Published:** 2022-05-10

**Authors:** Yueqin Chen, Lei Zhu, Hu Meng, Xiangdong Sun, Chunyan Xue

**Affiliations:** ^1^Department of Ophthalmology, Nanjing Drum Tower Hospital, The Affiliated Hospital of Nanjing University Medical School, Nanjing 210008, China; ^2^Department of Ophthalmology, Jinling Hospital, Nanjing University Medical School, Nanjing 210002, China; ^3^Department of Radiotherapy, Jinling Hospital, Nanjing University Medical School, Nanjing 210002, China

## Abstract

Ionizing radiation- (IR-) induced oxidative stress has been recognized as an important mediator of apoptosis in lens epithelial cells (LECs) and also plays an important role in the pathogenesis of IR-induced cataract. Ferulic acid (FA), a phenolic phytochemical found in many traditional Chinese medicine, has potent radioprotective and antioxidative properties via activating nuclear factor erythroid 2-related factor 2 (Nrf2) signal pathway. The goals of this study were to determine the protective effect of FA against IR-induced oxidative damage on human lens epithelial cells (HLECs) and to elucidate the role of Nrf2 signal pathway. HLECs were subjected to 4 Gy X-ray radiation with or without pretreatment of FA. It was found that FA pretreatment protected HLECs against IR-induced cell apoptosis and reduced levels of ROS and MDA caused by radiation in a dose-dependent manner. IR-dependent attenuated activities of antioxidant enzymes (SOD, CAT, and GPx) and decreased ratio of reduced GSH/GSSG were increased by pretreatment of FA. FA inhibited IR-induced increase of Bax and cleaved caspase-3 and the decrease of Bcl-2 in a dose-dependent manner. Furthermore, FA provoked Nrf2 nuclear translocation and upregulated mRNA and protein expressions of HO-1 in a dose-dependent manner. These findings indicated that FA could effectively protect HLECs against IR-induced apoptosis by activating Nrf2 signal pathway to inhibit oxidative stress, which suggested that FA might have a therapeutic potential in the prevention and alleviation of IR-induced cataract.

## 1. Introduction

Head and neck cancers are the seventh most common malignancy in the world with unacceptably high mortality rates, which is a significant public health burden worldwide [1]. Radiation therapy plays a key role in curative-intent treatments for these cancers. Despite advances in treatment delivery, fractionation schemas, and thoughtful interplay with technical surgical improvements, it is still burdened by a high rate of acute and late side effects [1]. Ionizing radiation- (IR-) induced cataract is a frequent and well-documented side effect in these patients [2].

Cataract, defined as opacification of the eye lens, is the leading cause of blindness in the world [3]. Numerous studies have demonstrated that oxidative stress plays a major role in the initiation and progression of cataract [4]. Ionizing radiation (IR) induces cell damage by generating reactive oxygen species (ROS) [5]. Lens epithelial cells (LECs) comprise the middle layer of the lens and are found to be the most metabolically active [6]. LECs are well equipped with antioxidant systems to combat oxidative stress [7]. When the ROS exceeds the antioxidant defensive protection in the LECs, it will result in oxidative damage which leads to apoptosis of LECs and cataract formation [4, 8]. At present, the only means to treat cataract is by surgical intervention. However, surgery may lead to some serious complications such as endophthalmitis, corneal edema, cystoid macular edema, and increased intraocular pressure, especially in the elderly people and in patients with systematic diseases [9, 10]. Therefore, it is necessary to develop nonsurgical therapeutics for the prevention and alleviation of IR-induced cataract.

Nuclear factor erythroid 2-related factor 2 (Nrf2) is one of the most important transcription factors that control the transcription of many antioxidant genes, such as glutathione reductase (GR), superoxide dismutase (SOD), GSH-Px (GPx), catalase (CAT), glutamate-cysteine ligase (GCL), and heme oxygenase-1 (HO-1), to combat ROS and boost cell survival [11, 12]. Our previous study has shown that IR-induced cataract is related to the incapacity of Nrf2 antioxidative systems [13]. Taken together, these findings suggest that Nrf2 inducers may serve as potential therapeutic agents for preventing or alleviating IR-induced cataract.

Ferulic acid (FA), a phenolic acid compound abundant in vegetables, fruits, grains, and some Chinese medicine, is able to scavenge ROS by increasing the activities of antioxidant enzymes via activating Nrf2 signal pathway [14–16]. Furthermore, many studies have demonstrated that FA is a nontoxic and effective radioprotectant in vivo and in vitro [17–19]. However, no data is available on the beneficial effect of FA on IR-induced cataract. Therefore, the present study was aimed at investigating the protective effect of FA against IR-induced oxidative stress in LECs and its underlying mechanisms and attempting to provide a novel agent for the prevention and alleviation of IR-induced cataract.

## 2. Materials and Methods

### 2.1. Materials and Chemicals

Fetal bovine serum (FBS) (#16000044) and 0.25% trypsin-EDTA (#2053591) solution were obtained from Gibco (Thermo Fisher Scientific, Inc.). FA (#LRAB6783) (purity >99.0%) was purchased from Sigma Chemical Co. (St. Louis, USA). Dulbecco's modified Eagle's medium (DMEM) (#KGM12800-500), dimethyl sulfoxide (DMSO) (#KGT5131), 4,5-dimethylthiazol-2-yl-2,5-diphenyltetrazolium bromide (MTT) (#KGA311), Annexin V-FITC Apoptosis Detection Kit (#KGA105), whole cell lysis assay kit (#KGP250), bicinchoninic acid (BCA) (#KGP902) protein quantitative detection kit, HRP-conjugated secondary antibody (#KGAA35), and chemiluminescence (ECL) detection kit (#KGP116) were bought from KeyGen Biotech Co. (Nanjing, China). Minute (TM) Cytoplasmic and Nuclear Fractionation kit (#SC-003) was from Invent Biotechnologies (MN, USA). MDA assay kit (#A003-1), total SOD assay kit (#A001-3), GPx assay kit (#A005-1-1), and CAT assay kit (#A007-1-1) were obtained from Jiancheng (Nanjing, China). GSH/GSSG assay kit (#S0053) was obtained from Beyotime Institute of Biotechnology (Shanghai, China). Anti-Nrf2 (# ab137550), anti-HO-1 (#ab189491), anti-Bax (#ab32503), anti-Bcl-2 (#ab182858), anti-procaspase-3 (#ab32150), anti-cleaved caspase-3 (#ab2302), anti-GAPDH (#ab9485), and anti-histone H3 (#ab1791) antibodies were bought from Abcam (Cambridge, UK). TRIzol reagent (#15596-026) was from Invitrogen (Waltham, USA). PrimeScript RT Master Mix (#RR036B) and One Step TB Green PrimeScript RT-PCR Kit II (SYBR Green) (#RR086B) were from Takara (Dalian, China).

### 2.2. Cell Culture

A human lens epithelial cell (HLEC) line (FHL124) was purchased from Binsui Biotech Co. (Shanghai, China). HLECs were cultured in DMEM supplemented with 10% FBS at 37°C in a humidified atmosphere of 5% CO_2_. Medium was replaced every 2 days, and the cells were digested with 0.25% trypsin-EDTA when the density of the cells reached 90%.

### 2.3. Cell Viability Assay

Cell viability was assessed using MTT assay. Briefly, HLECs were seeded in 96-well plates. After different treatment, 50 *μ*L MTT solution was added to each well and further incubated for 4 h to allow the conversion of MTT into formazan crystals. Subsequently, the medium was removed, and 150 *μ*l DMSO was added to each well to dissolve the crystals. The absorbance was measured at a wavelength of 490 nm using a microplate reader (Thermo Fisher Scientific, Inc.), and the cell survival ratio was expressed as a percentage of the control.

### 2.4. Treatment with FA and Irradiation Schedule

**Figure 1:**
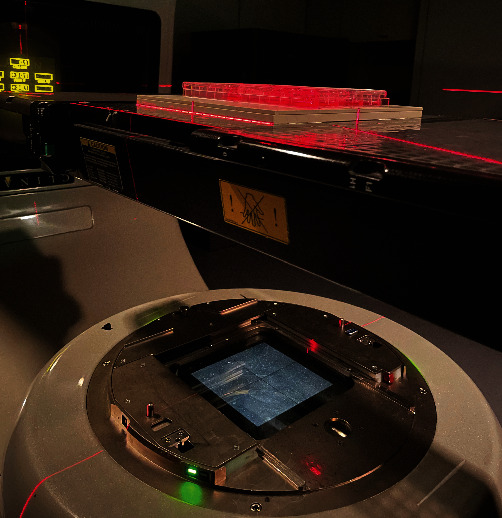
Diagram of the X-ray radiation of HLECs. Plates seeded with HLECs were placed in a 30 × 30 cm^2^ field on 1.5 cm of equivalent tissue material, and the radiation (4 Gy of X-ray) was delivered using Varian linear accelerator (Clinac-21EX, USA).

FA stock solution (0.5 mM) was prepared in DMSO. The working solution was diluted with DMEM so that the final concentrations of DMSO in the culture medium were not more than 0.01% (*v*/*v*). 0.01% DMSO was used as control. For cytotoxicity determination of FA, HLECs were exposed to different concentrations of FA (0, 50, 100, 150, 200, 250, 300, 350, 400, 450, and 500 *μ*M). Our preliminary MTT study showed that the concentrations of FA below 250 *μ*M had no significant toxicity on HLECs. To determine the protective effect of FA on IR-induced cell injury, HLECs were pretreated with FA (50, 100, and 200 *μ*M) for 2 hours followed by exposure to X-ray. The control cells were exposed to sham radiation. After radiation, the cells were subsequently incubated for 72 h. A single dose of 4 Gy (370 Mu) X-ray was delivered using Varian linear accelerator (Clinac-21EX, USA), when the 6-well plates were placed in a 30 × 30 cm^2^ field on 1.5 cm of equivalent tissue material (Figure 1).

### 2.5. Microscopic Observations of Cell Morphology

HLECs were seeded and incubated in 6-well plates for 24 h at 37°C. Further, the cells were treated with indicated concentrations of FA 2 h before radiation. Seventy-two hours after radiation, the cellular morphological changes were observed under phase contrast microscope (Olympus IX71, Japan) and photographed.

### 2.6. Flow Cytometry

The assessment of apoptosis was performed by using the Annexin V–FITC Apoptosis Detection Kit according to the manufacturer's protocol as described previously [20]. Briefly, after different interventions, the cells were harvested, washed with PBS, and resuspended in binding buffer. The cells (10^4^) were incubated with 5 *μ*l Annexin V-FITC and 5 *μ*l PI for 5 minutes in the dark at room temperature. Finally, the cells were analysed using flow cytometry (Becton-Dickinson, FACS Calibur, USA) to determine percentage of apoptosis.

### 2.7. Detection of Intracellular ROS

The level of ROS was monitored using the fluorescent probe 2,7-dichlorofluorescein diacetate (DCFH-DA) by flow cytometry as previously described [20]. Briefly, after different interventions, the cells (10^4^) were collected in each group and incubated with 10 *μ*M DCFH-DA at 37°C for 30 min in the dark. Then, the cells were washed twice using culture medium. The intracellular production of ROS was determined using flow cytometry (Becton-Dickinson, FACS Calibur, USA). The values of ROS levels were normalized to the control group and expressed as fold of the control.

### 2.8. Antioxidant Assay

After indicated interventions, the total cell extracts were prepared, and protein concentration was determined as previously described [21]. The activities of total SOD, CAT, and GPx, the content of MDA, and the ratio of reduced GSH/GSSG were measured using respective commercial diagnostic kits.

The activity for total SOD was examined according to xanthine oxidase method provided by the total SOD assay kit as described [22]. The assay used the xanthine/xanthine oxidase system to produce superoxide ions, which reacted with 2-(4-iodophenyl)-3-(4-nitrophenyl)-5-(2,4-disulfophenyl)-2H-tetrazolium to form a formazan dye, and the absorbance at 450 nm was determined. The values were expressed as units per milligram protein (U/mg protein), where one unit of SOD was defined as the amount of SOD inhibiting the rate of reaction by 50% at 25°C.

CAT activity was measured using a CAT kit based on the hydrolysis reaction of H_2_O_2_ with CAT, which could be terminated by molybdenum acid (MA) to produce yellow MA-H_2_O_2_ complex [23]. CAT activity was calculated by the decrease in absorbance at 405 nm due to the degradation of H_2_O_2_. The values were expressed as units per milligram protein (U/mg protein).

GPx activity was determined by the velocity method using a GPx kit [24]. The reaction was initiated by the addition of GPx in the cell extracts which subsequently led to the conversion of reduced GSH to oxidized glutathione (GSSG). The change in absorbance during the conversion of reduced GSH to GSSG was recorded spectrophotometrically at 412 nm. The values were expressed as units per milligram protein (U/mg protein).

Lipid peroxidation was evaluated by measuring MDA concentrations according to the thiobarbituric acid (TBA) method as commercially recommended [25]. The method was based on the spectrophotometric measurement of the colour produced during the reaction to TBA with MDA. MDA concentrations were calculated by the absorbance of TBA reactive substances (TBARS) at 532 nm. The concentration of MDA was expressed as nanomole per milligram protein (nmol/mg protein).

The reduced GSH/GSSG ratio was determined using a GSH/GSSG assay kit (Beyotime Institute of Biotechnology) according to the manufacturer's instructions [26]. The cell pellet was suspended in protein removal solution, thoroughly incorporated, and placed in -70°C and 37°C sequentially for fast freezing and thawing, then placed in 4°C for 5 min, and centrifuged at 10000 g for 10 min. The supernatant was used to determine the amount of glutathione in the sample. Total glutathione level was measured by the 5,5′-dithiobis (2-nitrobenzoic acid) (DTNB)-GSSG recycling assay. Briefly, GSH assay buffer, glutathione reductase, DTNB solution, and supernatant sample were mixed together and incubated at 25°C for 5 min, and then, NADPH was added into this system to trigger the reaction. The absorbance of TNB was measured at 412 nm, and the total glutathione level was calculated following the product instructions. GSSG was determined by the same method in the presence of 2-vinylpyridine. The amount of reduced GSH was obtained by subtracting the 2 × GSSG values from the total glutathione values.

### 2.9. Quantitative Real-Time PCR Analysis

Total RNA was extracted using TRIzol reagent following the manufacturer's protocol and reversely transcribed into cDNA using PrimeScript RT Master Mix (Takara, China). Real-time polymerase chain reaction (PCR) was carried out using an ABI Step one plus RT-PCR System (Applied Biosystems, USA) with specific primers and SYBR Green Master Mix (Takara, China). Both forward and reverse primers for target genes were provided by General Biol (Chuzhou, China), as shown in Table 1. The relative levels of mRNA were calculated using the 2^−*ΔΔ*CT^ method, normalizing for GAPDH and related to the control.

### 2.10. Western Blot

Whole cell protein lysates were extracted using a Whole Cell Lysis Assay Kit, and nuclear extracts were extracted using a Cytoplasmic and Nuclear Fractionation kit according to the manufacturer's instructions. Protein samples were loaded and separated on 10% SDS-PAGE and then transferred to polyvinylidene fluoride (PVDF) membranes and blocked with 5% skim milk for 2 h. After that, the membranes were incubated with primary antibodies (anti-Bax, anti-Bcl-2, anti-cleaved-caspase-3, anti-procaspase-3, anti-Nrf2, and anti-HO-1) overnight at 4°C. After the membranes were washed three times with TBST buffer, they were incubated in HRP-conjugated secondary antibody at room temperature for 1 h. The protein blots were visualized with ECL using G:BOX chemiXR5. The band densities of each sample were determined using ImageJ software.

### 2.11. Statistical Analysis

All experiments were performed in triplicate, and data were expressed as the mean ± SD. Statistical analysis was performed using a one-way analysis of variance (ANOVA) followed by LSD using SPSS software version 17.0. A value of *P* < 0.05 was considered to be statistically significant.

## 3. Results

### 3.1. Effects of X-Ray Radiation on Cell Viability of HLECs by MTT

**Figure 2:**
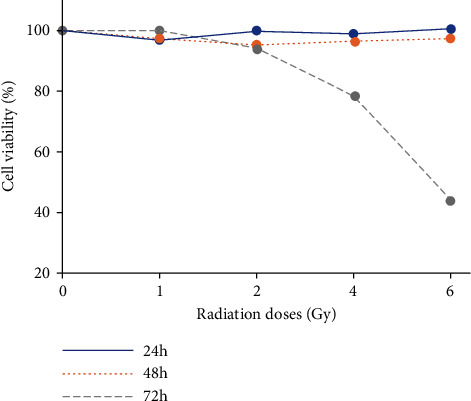
Cell viability of HLECs exposed to different doses of X-ray radiation at different time by MTT. HLECs were exposed to 0, 1, 2, 4, and 6 Gy X-ray radiation and then incubated for 24, 48, and 72 h.

To evaluate the effects of radiation on cell viability, HLECs were exposed to 0, 1, 2, 4, and 6 Gy X-ray radiation and then observed for 24, 48, and 72 h. As shown in Figure 2, cell viability was unchanged under different doses radiation at 24 and 48 h (*P* > 0.05), and cell viability began to decrease at 4 Gy at 72 h (*P* < 0.05). Therefore, we developed the radiation model by exposing the cells under 4 Gy X-ray radiation and then incubation for 72 h.

### 3.2. The Cytotoxicity of FA on HLECs by MTT

**Figure 3:**
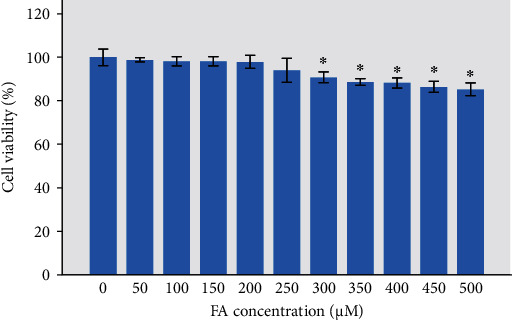
Cell viability of HLECs treated with different concentrations of FA by MTT. HLECs were incubated with FA for 72 h. ^∗^*P* < 0.05 vs. the control group.

To explore the cytotoxicity of FA on HLECs, cell viability was evaluated by MTT after they were exposed to different concentrations (0, 50, 100, 150, 200, 250, 300, 350, 400, 450, and 500 *μ*M) of FA for 72 h. As shown in Figure 3, the concentrations of FA below 250 *μ*M had no significant toxicity on HLECs (*P* > 0.05). Therefore, 50, 100, and 200 *μ*M FA were used for further experiments.

### 3.3. FA Alleviated IR-Induced Cell Morphological Change in HLECs

**Figure 4:**
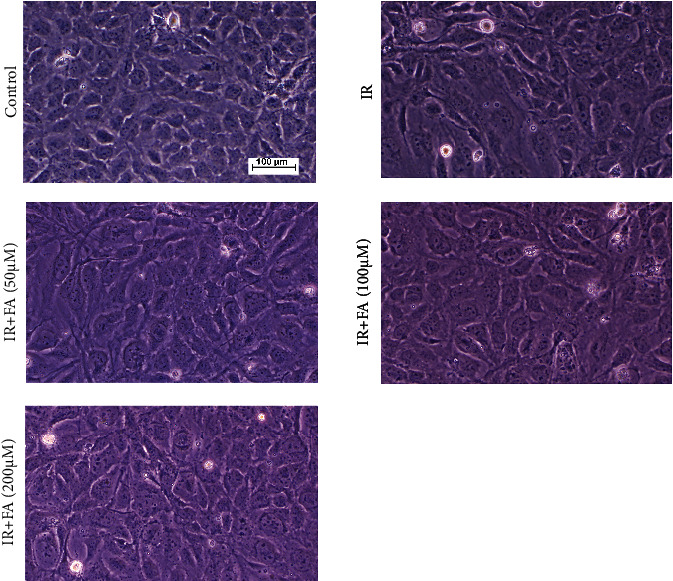
The effect of FA on IR-induced cell morphological changes in HLECs (10×). Representative images of cell morphology in each group. HLECs were pretreated with FA (50, 100, and 200 *μ*M) for 2 hours followed by exposure to 4 Gy X-ray radiation and then incubated for 72 h.

To evaluate the protective effect of FA on IR-induced damage in HLECs, the cells were pretreated with FA (50, 100, and 200 *μ*M) for 2 hours followed by exposure to a single dose of 4 Gy X-ray. After incubation for 72 h, we observed the cell morphology under phase contrast microscope. As shown in Figure 4, HLECs displayed epithelial cell-like adherent growth and clear outline and were tightly arranged in the control. After radiation, the cells were swollen and disorderly arranged with fuzzy outline. Pretreatment with FA improved cell morphological changes caused by radiation. However, cell morphology did not return to normal.

### 3.4. FA Reduced IR-Induced Cell Apoptosis in HLECs

**Figure 5:**
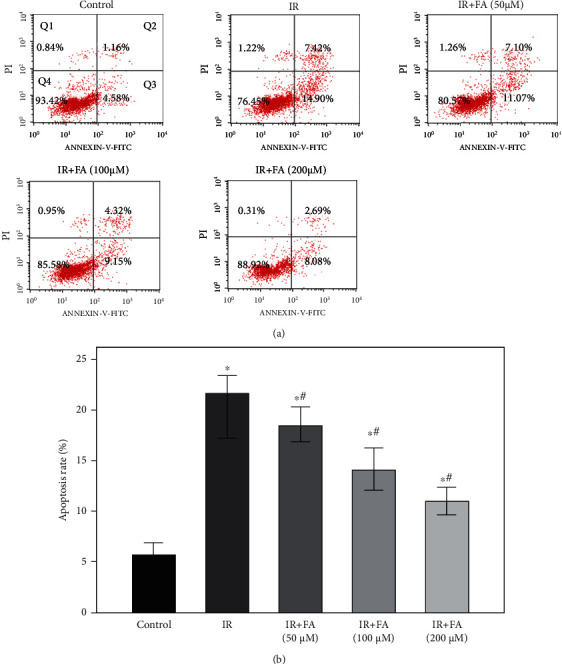
Protective effect of FA against IR-induced cell apoptosis in HLECs. (a) Flow cytometry analysis of cell apoptosis using Annexin V-FITC/PI dual staining. (b) Quantitative results of the apoptosis rate. Annexin V-positive cell (Q2+Q3) was calculated for each group cells and is shown in the bar graph. HLECs were pretreated with FA (50, 100, and 200 *μ*M) for 2 hours followed by exposure to 4 Gy X-ray radiation and then incubated for 72 h. All data were expressed as means ± SD. ^∗^*P* < 0.05 vs. the control group; ^#^*P* < 0.05 vs. the IR group.

To investigate whether FA protected against IR-induced apoptosis, flow cytometric analysis was performed to quantify the rate of cell apoptosis using double staining of Annexin V-FITC and PI. As shown in Figure 5, a significant increase of apoptosis was observed in HLECs after 4 Gy X-ray radiation compared with the control (*P* < 0.05). However, FA pretreated HLECs showed resistance to IR-induced apoptosis in a dose-dependent manner (*P* < 0.05).

### 3.5. FA Regulated IR-Induced Apoptosis-Related Protein Expression in HLECs

**Figure 6:**
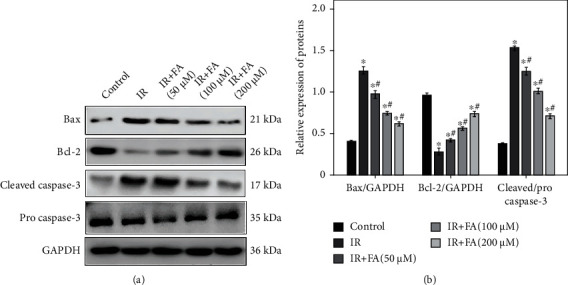
The effect of FA pretreatment on IR-induced changes of Bax, Bcl-2, cleaved caspase-3, and procaspase-3 in HLECs. (a) Representative blots of Bax, Bcl-2, cleaved caspase-3, procaspase-3, and GAPDH. (b) Bar graphs of relative protein expression of Bax, Bcl-2, and caspase-3 in each group. HLECs were pretreated with FA (50, 100, and 200 *μ*M) for 2 hours followed by exposure to 4 Gy X-ray radiation and then incubated for 72 h. All data were expressed as means ± SD. ^∗^*P* < 0.05 vs. the control group; ^#^*P* < 0.05 vs. the IR group.

To further confirm the inhibitory effect of FA on IR-induced apoptosis, we detected the protein expressions of Bax, Bcl-2, and pro and cleaved caspase-3 in HLECs by Western blot.  Figure 6 showed that a significant increased Bax expression and cleaved/procaspase-3 level occurred in HLECs after exposure to radiation, in combination with a decreased Bcl-2 expression. FA pretreatment for 2 hours intensely downregulated Bax expression and cleaved/procaspase-3 level and upregulated the Bcl-2 expression as compared to IR group in a dose-dependent manner (*P* < 0.05).

### 3.6. FA Suppressed IR-Induced ROS and Lipid Peroxidation in HLECs

**Figure 7:**
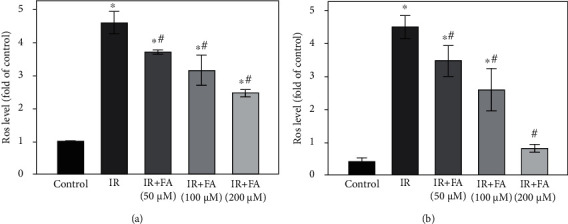
The effect of FA pretreatment on IR-induced ROS and lipid peroxidation in HLECs. (a) The effect of FA pretreatment on IR-induced change of ROS in HLECs. (b) The effect of FA pretreatment on IR-induced change of MDA in HLECs. HLECs were pretreated with FA (50, 100, and 200 *μ*M) for 2 hours followed by exposure to 4 Gy X-ray radiation and then incubated for 72 h. All data were expressed as means ± SD. ^∗^*P* < 0.05 vs. the control group; ^#^*P* < 0.05 vs. the IR group.

ROS and MDA (a lipid peroxidation product) levels were assayed to evaluate oxidative stress induced by IR and the protective effect of FA. ROS, measured using DCFH-DA, increased about four times after 4 Gy X-ray radiation as compared to the control, while pretreatment with FA reduced IR-induced ROS production in a dose-dependent manner (*P* < 0.05) (Figure 7(a)).

Exposure of HLECs to X-ray radiation led to a significant increase in MDA compared to the control, whereas pretreatment with FA protected the cells against lipid peroxidation in a dose-dependent manner (*P* < 0.05) (Figure 7(b)).

### 3.7. FA Prevented IR-Induced Decreases in Antioxidative Enzymatic Activities and Ratio of Reduced GSH/GSSG in HLECs

**Figure 8:**
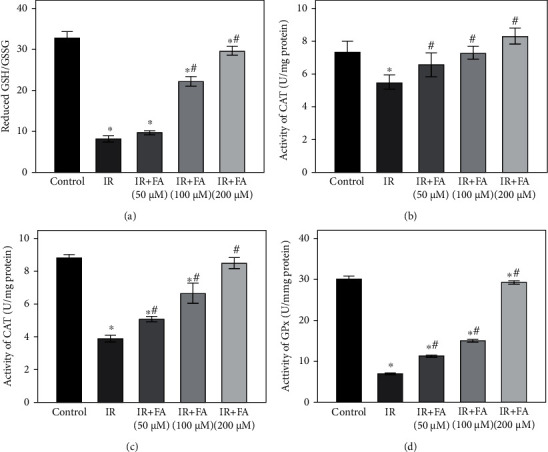
The effect of FA pretreatment on antioxidant status in HLECs. The effect of FA pretreatment on the ratio of reduced GSH/GSSG (a) and activities of T-SOD (b), CAT (c), and GPx (d) in HLECs. HLECs were pretreated with FA (50, 100, and 200 *μ*M) for 2 hours followed by exposure to 4 Gy X-ray radiation and then incubated for 72 h. All data were expressed as means ± SD. ^∗^*P* < 0.05 vs. the control group; ^#^*P* < 0.05 vs. the IR group.

To clarify whether the protective effect of FA was related to the alteration of intracellular redox status, we examined intracellular reduced GSH/GSSG level and activities of T-SOD, CAT, and GPx in HLECs. As shown in Figure 8, intracellular reduced GSH/GSSG level and activities of SOD, CAT, and GPx were significantly downregulated by radiation; pretreatment with FA provided significant protection against downregulation of intracellular reduced GSH/GSSG level and activities of SOD, CAT, and GPx caused by radiation in a dose-dependent manner (*P* < 0.05).

### 3.8. FA Upregulated the Nrf2/HO-1 Signal Pathway in HLECs

**Figure 9:**
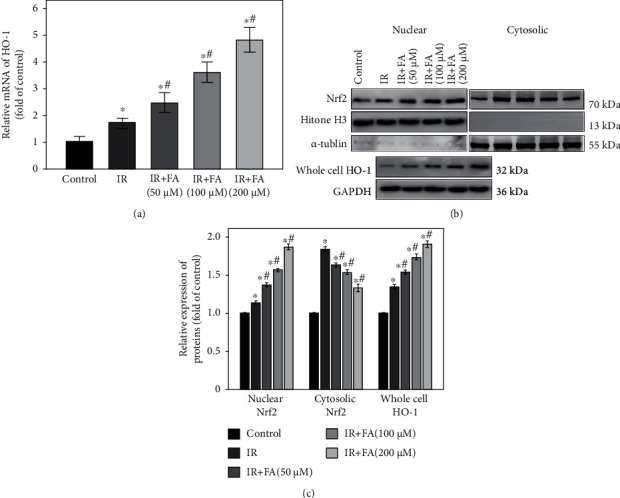
Pretreatment of FA upregulated Nrf2 signal pathway in IR-induced HLECs. (a) Bar graph of mRNA expression of HO-1 determined by qRT-PCR. (b) Representative blots of nuclear Nrf2, cytosolic Nrf2, Histone H3, *α*-tubulin, HO-1, and GAPDH. (c) Bar graph of protein expressions of nuclear Nrf2, cytosolic Nrf2, and whole cell HO-1. HLECs were pretreated with FA (50, 100, and 200 *μ*M) for 2 hours followed by exposure to 4 Gy X-ray radiation and then incubated for 72 h. Data were expressed as mean ± SD. ^∗^*P* < 0.05 vs. the control group; ^#^*P* < 0.05 vs. the IR group.

To determine the molecular mechanism of FA on antioxidant capability, the effect of FA on Nrf2 nuclear translocation and its downstream antioxidant gene in IR-induced HLECs was examined. As shown in Figure 9, treatment with FA before radiation significantly increased nuclear Nrf2 and decreased cytosolic Nrf2 in a dose-dependent manner compared with IR group (*P* < 0.05). Interestingly, we found that the levels of nuclear and cytosolic Nrf2 were both increased in IR group compared to the control group which might be mainly related with oxidative stress (*P* < 0.05). In addition, we found that mRNA and protein levels of HO-1 were upregulated upon radiation, and FA could further augment its expression (*P* < 0.05).

## 4. Discussion

The lens is one of the most radiosensitive tissues in the human body, and it is known that ionizing radiation can induce cataract, which is ranked second among occupational diseases associated with radiation in China [27]. Our previous study showed that IR-induced cataract is related to the incapacity of Nrf2 antioxidative systems in the lens [13]. Enhanced oxidative stress and decreased antioxidant defensive capacity are thought to be the two main contributors to the pathogenesis of cataract [28]. LECs have powerful defensive mechanisms against oxidation, with Nrf2 as a vital one, which can eliminate ROS and maintain the redox balance [29]. Nrf2 is normally sequestered in the cytoplasm; upon activation by ROS, its level rises and translocates into the nucleus to bind to ARE, which in turn upregulates the expression of downstream antioxidant genes, such as HO-1, to combat ROS and boost cell survival [30].

There have been increasing interests in developing natural antioxidant nutrients that could effectively prevent and delay the formation of cataract [31–33]. FA, a phenolic acid, can be separated from many medicinal and edible plants and has been shown to have antioxidant and radioprotective activities [20, 34]. And these effects are related to its ability to scavenge ROS by increasing the activities of antioxidant enzymes via activating Nrf2 signal pathway [34, 35]. The current study appears to be the first in the literature showing the antioxidant and radioprotective effect of FA on IR-induced oxidative damage in HLECs.

IR-induced damage is primarily attributed to ROS, which plays a critical role in the occurrence and development of IR-induced cataract [4]. Nevertheless, LECs have antioxidant defensive systems to scavenge ROS, including nonenzymatic and enzymatic antioxidant defensive mechanisms [7]. GSH, the main nonenzymatic antioxidant in LECs, protects cells against ROS; reduced GSH converts to its oxidized form (GSSG) when it reacts with ROS and is reinstated through GR action [28]. SOD, CAT, and GPx are the main enzymatic antioxidants in LECs. SOD can transform O2^−^ into H_2_O_2_ and molecular oxygen, whereas the CAT and GPx convert H_2_O_2_ into water [36, 37]. In this way, two toxic species, O^2−^ and H_2_O_2_, are converted to the harmless product water. When the activities of these enzymatic antioxidants increase, the capability of eliminating ROS enhances. Conversely, a decrease in these protective mechanisms' activity may result in elevated ROS which can cause membrane lipid peroxidation, breakdown of transmembrane ion gradients, and loss of cellular viability and ultimately lead to LECs damage and even death [38]. One of the by-products of lipid peroxidation is the toxic compound malondialdehyde (MDA), whose involvement in cataractogenesis has been suggested [6]. Ma et al. reported that FA pretreatment significantly attenuated IR-induced ROS production and provided significant protection against downregulation of GSH in HUVECs in a dose-dependent manner [39]. El-Mesallamy et al. found that pretreatment with FA before radiation exhibited a marked protection against IR-induced oxidative stress and conserved levels of MDA, GSH, and SOD activity toward the control values in testis tissues of rats [19]. Our data suggested that FA pretreatment could protect HLECs against IR-induced decrease of reduced GSH/GSSG level and compromised activities of SOD, CAT, and GPx, attenuated IR-induced ROS production and lipid peroxidation, and protected IR-induced oxidative damage. Our results were similar to Ma et al. and El-Mesallamy et al.'s.

IR-induced oxidative stress can cause cell apoptosis [36]. Bax and Bcl-2 are the key regulators of cell apoptosis. IR has been reported to be able to increase proapoptotic member Bax and decrease antiapoptotic member Bcl-2 gene expression [36]. The imbalance between Bax and Bcl-2 can induce mitochondrial membrane permeabilization and then cause the release of Cyt c from the mitochondria to activate the caspase cascade. The upregulation of cleaved caspase-3 is the final executioner enzyme of apoptosis [40]. In our study, X-ray radiation indeed resulted in significant apoptosis in HLECs and augmented the expression of Bax and cleaved caspase-3 while decreased Bcl-2. Besides, we found that pretreatment of FA increased Bcl-2, decreased Bax and cleaved caspase-3 level, and alleviated IR-induced apoptosis of HLECs. A number of studies have also demonstrated the protective effect of FA on IR-induced cell apoptosis by regulating Bax, Bcl-2, and cleaved caspase3 expression [41].

Nrf2 signal pathway is highly associated with oxidative stress in the lens [13, 42]. Nrf2 has been proved to be maintained at a low level through 26S proteasome-mediated degradation and Keap1-mediated ubiquitylation. However, Nrf2 is stabilized, and its level rises when the cells are exposed to electrophiles or oxidants [43]. So as in our study, Nrf2 was released and stabilized, and its level was increased after HLECs exposed to radiation. Upon oxidative insult, stabilized Nrf2 can be translocated into the nucleus, followed by the upregulation of its downstream antioxidant genes to combat oxidative stress. That is why both nuclear and cytosolic Nrf2 and its downstream gene HO-1 were augmented upon radiation in our study. However, when the oxidative stress overwhelms the intrinsic antioxidant capacity in cells, it results in cell damage [39, 42]. This may explain our results that although Nrf2 signal pathway was activated upon radiation, nevertheless, HLECs still suffered oxidative damage. There are many studies demonstrating that FA can improve Nrf2 nuclear translocation and upregulate its downstream antioxidant genes, such as HO-1, to protect cells against oxidative stress [34, 44, 45]. HO-1, a stress protein, has been shown to be able to counteract oxidative injury induced by ROS. Abraham et al. transfected the human HO-1 gene into rabbit ocular tissues in vivo and found that it could be a promising means to protect against oxidative stress-related ocular diseases, such as cataract [46]. In the present study, our results showed that pretreatment with FA further augmented the nuclear translation of Nrf2 and upregulated the expression of HO-1 to protect HLECs against radiation-induced oxidative damage.

## 5. Conclusions

In summary, the present study demonstrated that FA protected HLECs against IR-induced oxidative damage by upregulating Nrf2/HO-1 antioxidant signal pathway to increase the capacity to scavenge ROS. Our findings provide theoretic basis and experimental evidence for the application of FA in the prevention and alleviation of IR-induced cataract. However, further studies are needed to verify the radioprotective effects of FA on IR-induced cataract and the role of Nrf2/HO-1 signal pathway in an *in vivo* model of cataract.

## Figures and Tables

**Table 1:** Primers used for qRT-PCR.

Gene	Primer sequences (5′→3′)
HO-1 (NM_002133)	F: AAGACTGCGTTCCTGCTCAAC
	R: AAAGCCCTACAGCAACTGTCG
GAPDH (NM_001256799)	F: AAAGCCTGCCGGTGACTAAC
	R: AGGAAAAGCATCACCCGGAG

## Data Availability

The data used to support the findings of this study are available from the corresponding author upon request.
